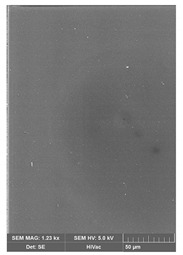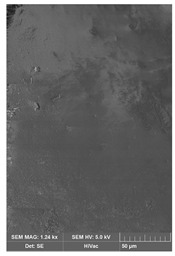# Selective Transport of Ag(I) through a Polymer Inclusion Membrane Containing a Calix[4]pyrrole Derivative from Nitrate Aqueous Solutions

**DOI:** 10.3390/ijms21155348

**Published:** 2020-07-28

**Authors:** Anna Nowik-Zajac, Iwona Zawierucha, Cezary Kozlowski

**Affiliations:** Institute of Chemistry, Jan Dlugosz University of Czestochowa, PL42200 Czestochowa, Poland; i.zawierucha@ajd.czest.pl (I.Z.); c.kozlowski@ajd.czest.pl (C.K.)

**Keywords:** calix[4]pyrroles, polymer inclusion membranes, silver ions, selective transport

## Abstract

Cellulose-triacetate-based polymer inclusion membranes (PIMs) with different concentrations of a calixpyrrole ester derivative as the membrane carrier were studied to determine their ability to transport Ag(I) from aqueous nitrate solutions. The effects of the concentrations of ion carriers and metal ions, the pH of the source aqueous phase, and stripping agents on the effective transport of Ag(I) were assessed. All studied parameters were found to be important factors for the transport of Ag(I) metal ions. The initial fluxes were determined at different temperatures. The prepared membranes were found to be highly permeable. The selectivity of silver transport from an aqueous solution containing Ag(I), Cu(II), Pb(II), Cd(II), Ni(II), Zn(II), and Co(II) ions was also investigated.

## 1. Introduction

Separation techniques for removing and recovering metals from liquid industrial waste are very important, because of the growing environmental protection problem [1]. Recycling of pure precious metals, including silver, brings many measurable benefits, both ecological (reduction of pollution, protection of natural resources) and economic (stabilization of metal prices on the market) [2]. Silver, as the most useful precious metal, is used in many applications and areas, especially in electronics, jewellery, photography, and alloys. The use of silver in industry and medicine for the production of alloys, photographic emulsion, and drugs has led to increasing interest in this metal [3].

In global production, above ten percent of silver sources are used to manufacture photosensitive units. A large part of silver is removed along with waste from the photographic and galvanizing industry [2]. Silver is also a toxic ion; therefore, the recovery of this metal from industrial wastewater currently has an ecological aspect [4]. Silver can be emitted to the environment as metallurgy water, because it usually occurs as a pollutant in cooper, zinc, and arsenic ores [2]. Being precious and toxic, silver must be recovered and separated from industrial effluents [5].

Different techniques were used for the removal of silver ions from aqueous solutions, such as solvent extraction [6,7,8], adsorption [9,10], emulsion liquid membrane (ELM) [11,12], bulk liquid membrane (BLM) [13,14], supported liquid membrane (SLM) [15,16,17], and polymer inclusion membrane (PIM) [18,19].

Solvent extraction and other industrial separation methods are sometimes not suitable because they are time-consuming, have high equipment cost, and produce toxic sludge [20]. In recent years, interest in macrocyclic compounds such as crown ethers, calixarenes, calixpyrolles, or cyclodextrins and their derivatives as potential metal ion carriers has been associated with their complex-forming properties. Calixarenes, especially their derivatives, functionalized with ionizable groups, e.g., ester groups, proved to be very versatile carriers of both neutral molecules and metal ions [21].

Maming et al. [22,23] studied the transport of Pb(II), Cd(II), and Ag(I) ions through bulk liquid membranes (BLM) containing *p-tert*-butylcalix[4]arene-tetraethylester. This ion carrier was selective for Ag(I) with separation factors for Cd(II) and Pb(II) over Ag(I) equal to 16.00 and 42.8, respectively.

As examples of macrocycles, calixpyrroles are of great interest for metal ion selectophores, due to their ready synthetic approaches, often high yields, and easy modification of their complexing properties through functionalization via the introduction of appropriate substituents. It was found that non-functionalized calixpyrroles could not be used for heavy metal ion separation, due to their low selectivity and low transport fluxes; therefore, considerable modification is necessary. The drawback of neutral calixpyrroles is their selectively low transport rate and lack of possibility for control of the transport process through pH changes in aqueous solution [24]. This drawback does not exist in ionisable calixpyrroles, e.g., ester or carboxylic ionic groups.

Calixpyrroles containing ester groups often bind to cations through cooperative interaction of cations with the calixpyrrole cavity and the ionic (ester) group. The pH change results in the disappearance of ionic interaction, i.e., the cation becomes free; this behaviour enables the use of the above compounds as ion carriers. The transfer mechanism is not exactly related to the co-transport mechanism, i.e., the ion pair is released due to ring opening, which is influenced by pH change [24].

Functionalized calix[4]pyrroles containing carboxylic or ester groups as well as derivatives of calix[4]pyrrole[2]thiophene can be used for efficient and selective removal of Ag(I) from a dilute aqueous solution containing Cu(II), Zn(II), and Cd(II), by transport through the polymer inclusion membranes [25].

Kaledkowski et al. [26] synthesized calix[4]pyrrole[2]thiophene, crosslinked in the form of a copolymer chelating the metal ions. Sorption studies of this material showed its high affinity to noble metal ions such as Ag(I), Au(III), Pt(IV), and Pd(II), while its affinity toward Pb(II) and Tl(I) ions is lower. The high selectivity of calix[4]pyrrole[2]thiophene toward Ag(I), Au(II), Pt(IV), and Pd(II) results from the presence of soft electrodonating sulfur atoms, which strongly interact with soft noble metal cations. Amiri et al. [2] reported the selective transport of Ag(I) through liquid membranes with calix[4]pyrroles as the ion carrier. It was shown that membranes containing calix[4]pyrroles have selectivity for silver over divalent transition metal ions. Moreover, Jain et al. [27] found higher sorption activity of immobilized calix[4]pyrrole[2]thiophene-derived resins toward Cu(II), Zn(II), and Cd(II) ions than that of commercial resins. Nowik-Zajac et al. [18] studied Ag(I) and Cu(II) ion transport across PIMs containing calix[4]pyrrole[2]thiophene as an ion carrier and established that the transport of Ag(I) ions is faster than that of Cu(II) ions. The selectivity of this investigated membrane system is determined by the percolation process of membrane components. Kolodziejska et al. [19] found that polymer inclusion membranes containing N-(diethylthiophosphoryl)aza[18]crown-6 and N-(diethyloxophosphoryl)-aza[18]crown-6 as carriers, act efficiently with silver ions. When the metal ions are inserted deeper in the ether cavity, the selectivity coefficient of Ag(I)/Cu(II) is higher. This was observed for the case of carrier concentration influence, partly due to differences in the density of charge of these metal cations and the varied stoichiometry of the association between the metal cations and the complexes. Nowik-Zajac et al. [28] studied silver transport through PIM with a carboxyl derivative calix[4]pyrrole. The transport described in this study was effective when the pH of the source phase was above 4.0.

The literature review shows several applications of liquid membranes to the treatment of real waste solutions. According to the literature [2,29,30], the concentration of Ag(I) ions in wastewater is between 4.0 and 8.0 × 10^−4^ M. Tarahomi et al. [29] showed the composition of silver plating and photographic waste solution in which the silver concentration was 5 × 10^−4^ M at pH 3. Amiri et al. [2] also showed that the derivatives of the calix[4]pyrroles dissolved in oil and immobilized to the supported liquid membrane (SLM), based on a polypropylene matrix, are effective and selective Ag(I) ion transporters. The concentration of Ag(I) ions was found to be 8.0 × 10^−4^ M, pH 4. In the following experiments, the silver concentration in the source aqueous phase was 1 × 10^−3^ M at pH 4. 

In this study, the transport of Ag(I) from the source solution to the receiving solution was performed using ester derivative calix[4]pyrrole-based plasticizer membranes. For this purpose, we studied the effects of different parameters, like the metal concentration and the types of receiving phases, on the transport process of silver ions from the source solutions prepared at various pH levels. We also investigated the following parameters—the effect of the carrier concentration and plasticizer content, and, especially, the effect of temperature and diffusion of carrier complexes on the transport processes.

## 2. Results and Discussion 

### 2.1. Modification of Source Phase Composition

**Figure 1:**
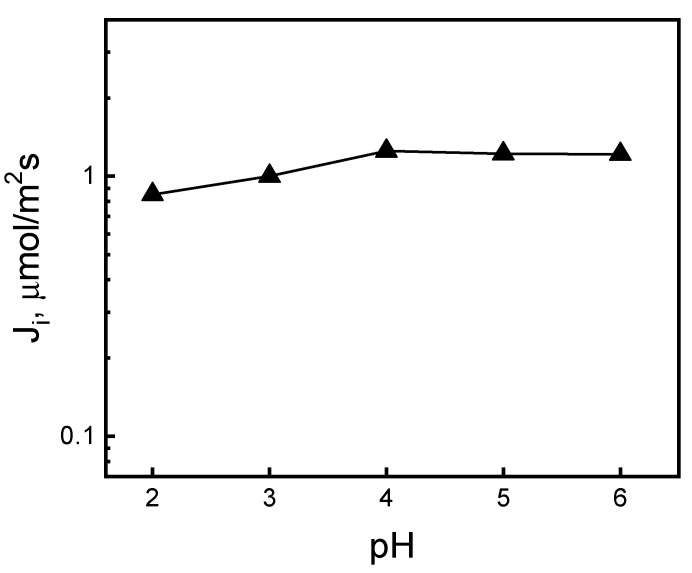
The relationship of the initial ion flux of Ag(I) transport across the PIM vs. the pH of the source phase. Source phase: 5.0 × 10^−4^ M AgNO_3_; membrane: 2.0 cm^3^ *o*-NPPE/1.0 g CTA; 0.050 M KP, receiving phase: 0.10 M thiourea.

The effect of source phase acidity within a pH range of 2.0 to 6.0 on Ag(I) ion transport through polymer inclusion membranes was examined. Figure 1 presents the relationship of initial fluxes of Ag(I) ions transport on the pH of the source phase containing 5.0 × 10^−4^ M AgNO_3_ through PIM, to the receiving phase containing 0.10 M of thiourea.

As was indicated, values of the initial fluxes of Ag(I) ion transport increased with an increasing pH from 2.0 to 4.0. For pH = 4.0, the maximum initial flux of Ag(I) ion transport from the aqueous source phase containing 5 × 10^−4^ M AgNO_3_ through PIM amounted to 1.25 µmol/m^2^s for KP. However, in the pH range of 4.0–6.0, the transport flux was stabilized at a constant level. Figure 1 shows that the acidity of the aqueous phase does not significantly affect the rate of Ag(I) transport by PIM containing an ester derivative calix[4]pyrrole. This was contrast with our previous study concerning Ag(I) transport by PIM containing a carboxyl derivative calix[4]pyrrole [28].

**Figure 2:**
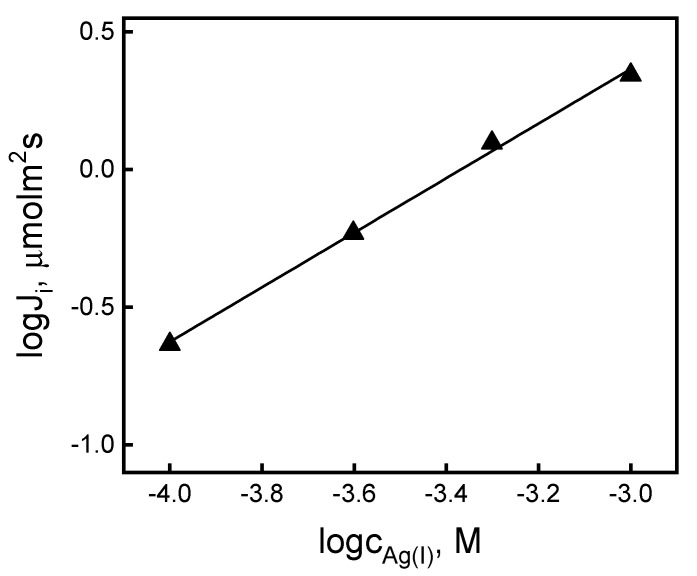
Logarithmic relationship of initial fluxes vs. Ag(I) concentration in the source aqueous phase. Source phase—various concentrations of AgNO_3_, pH = 4.0; membrane: 2.0 cm^3^ *o*-NPPE/1.0 g CTA; 0.050 M KP, receiving phase: 0.10 M thiourea.

Then, the effect of the initial AgNO_3_ concentration in the source phase on the rate of transport through PIM was examined. Four AgNO_3_ solutions at pH 4.0, containing 1.0 × 10^−4^ M, 2.5 × 10^−4^ M, 5.0 × 10^−4^ M, and 1.0 × 10^−3^ M of metal ions were prepared, and they were used as the source phases, in order to determine the kinetics of the PIM process due to the concentration of Ag(I) ions in the source phase. The 0.10 M thiourea solution was used as the receiving phase. Figure 2 shows the obtained linear relationships of (*J_i_*) for the Ag(I) concentration in the source phase in a log–log arrangement.

The change in the values of the initial Ag(I) flux as a function of the metal concentration in the source phase (*c_i_*) in a logarithmic arrangement was linear. This indicated that the reactions taking place in the boundary of the source phase/membrane involve a single Ag(I) cation. 

### 2.2. Modification of Receiving Phase Composition

**Figure 3:**
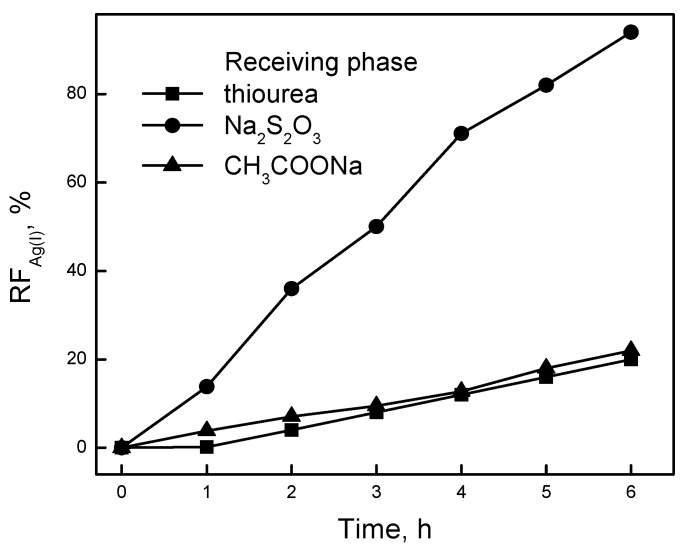
The removal factor (*RF*) obtained for the transport of Ag(I) ions across the polymer inclusion membrane (PIM). Source phase—1.0 × 10^−3^ M AgNO_3_, pH = 4.0; membrane: 2.0 cm^3^ *o*-NPPE/1.0 g CTA; 0.050 M KP, receiving phase—0.10 M Na_2_S_2_O_3_; 0.10 M thiourea and 0.10 M CH_3_COONa.

Aqueous solutions of thiourea, sodium thiosulfate (Na_2_S_2_O_3_), and sodium acetate (CH_3_COONa) were used to determine the influence of the receiving phase on the rate of transport at the boundary of the membrane/receiving phase. Figure 3 presents the relationship of the removal factor (*RF*) with the time taken for Ag(I) ion transport through PIM containing KP. As the receiving phases, we used aqueous solutions of 0.10 M thiourea, 0.10 M Na_2_S_2_O_3_, as well as 0.10 M CH_3_COONa at pH = 7.3.

The maximum value obtained for KP for the Ag(I) removal factor after 6 h of transport was equal to *RF* = 92.77% (0.10 M Na_2_S_2_O_3_ as the receiving phase). The yield of Ag(I) ion separation using the above-mentioned receiving phases was subject to a decrease in the following order: Na_2_S_2_O_3_ >> CH_3_COONa > thiourea. A total of 0.10 M of Na_2_S_2_O_3_ solution proved to be an effective compound for the decomplexation of the labile Ag(I)–calixpyrroles complex in the membrane. The separation of Ag(I) ions from aqueous solutions using CH_3_COONa and thiourea was low and did not exceed the value *RF* = 11%. Maming et al. [22] also suggested a Na_2_S_2_O_3_ solution as an appropriate receiving phase in the membrane system used for the selective Ag(I) removal. They demonstrated that PIM containing *p*-*tert*-buthylcalix[4]-arene-tetraethyl ester gives the most efficient transport for Ag(I) ions when 0.10 M od the Na_2_S_2_O_3_ solution was used as the receiving phase. The removal factor for Ag(I) ions was 65.9%. Using 0.15 M Na_2_S_2_O_3_ as the receiving phase, Amiri et al. [2] determined the rate and efficiency of the transport of Ag(I) ions through PIM containing calix[4]pyrrole. Then, the flux and *RF* for Ag(I) were found to be equal to 1.57 µmol/m^2^s and 38%, respectively.

### 2.3. Modification of Membrane Composition—The Effect of the Ion Carrier Concentration

An important factor affecting the efficiency of the separation of metal ions by transport through polymer inclusion membranes is the composition of the membrane phase, i.e., the type and concentration of the ion carrier, the type and amount of plasticizer, and the type and thickness of the polymer matrix used.

The effects of the type and quantity of the ion carriers derived from calixpyrroles in the polymer inclusion membrane on Ag(I) ion permeation were examined. Membranes with fixed contents of CTA (25 mg) and plasticizer (2 cm^3^ *o*-NPPE/1.0 g CTA) were prepared, while the concentration of the carrier in the membrane was changed in the range of 0.0010–0.10 M (based on the plasticizer volume) Each experiment was carried out for 6 h. The membranes without an ion carrier did not transport Ag(I) ions, which indicated that the carrier concentration is crucial for the facilitated transport of silver ions through PIM.

**Figure 4:**
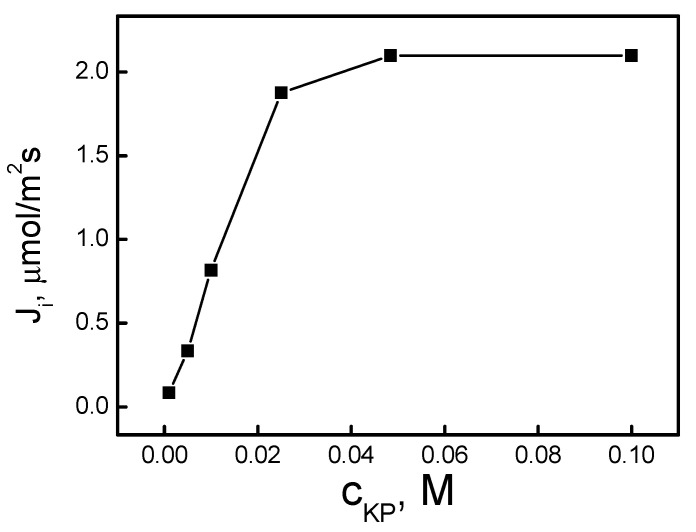
The Ag(I) transport flux vs. the ion carrier concentration in the PIM. Source phase: 1.0 × 10^−3^ M AgNO_3_, pH = 4.0; membrane: 25 mg CTA; 2.0 cm^3^ *o*-NPPE/1.0 g CTA; receiving phase: 0.10 M Na_2_S_2_O_3_.

Figure 4 shows that the transport efficiency increased sharply (linearly in the log–log system) with an increasing KP concentration from 0.0010 up to 0.050 M, where the yield of the metal removal reached 97.8%. Above 0.050 M KP, the yield of Ag(I) transport was close to 98%, within the experimental error, and the initial flux of Ag(I) seemed to reach a plateau.

The saturation of the polymer inclusion membrane with the ion carrier occurred at a membrane KP carrier concentration equal to 0.050 M (based on plasticizer volume). The rate of transport determined for that concentration of the carrier was maximal and reached 2.25 µmol/m^2^s.

This phenomenon is described in [31,32] and can be explained by an increase in the viscosity of the organic membrane phase and thus an increase in the resistance of the membrane, limiting complex diffusion through the membrane [33], or by a change in the transport mechanism from diffusion to jumping, induced by carrier crystallization within the membrane [32].

### 2.4. An Effect of the Membrane Plasticizer

Then, the effect of the type and concentration of the plasticizer in the polymer inclusion membrane on Ag(I) transport from the source phase at a concentration of 1.0 × 10^−3^ M and pH = 4.0 to 0.10 M Na_2_S_2_O_3_ solution as the receiving phase was examined.

**Figure 5:**
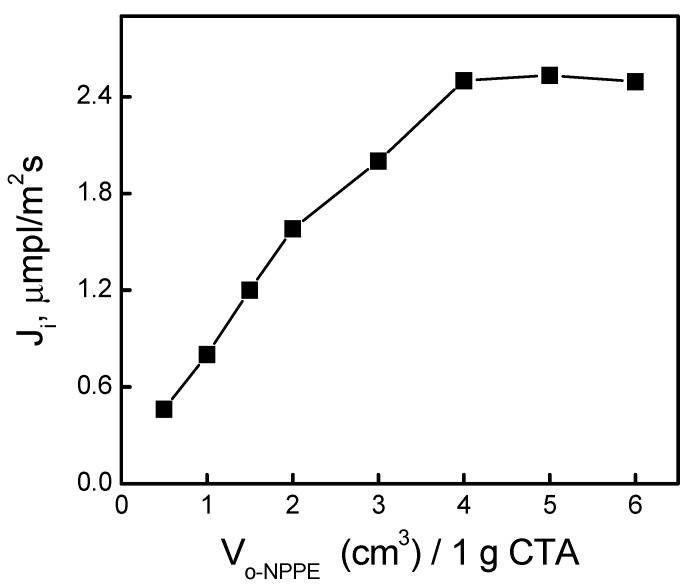
The effect of *o*-NPPE plasticizer on Ag(I) transport through a PIM containing KP. Source phase: 1.0 × 10^−3^ M AgNO_3_, pH = 4.0; membrane: 25.0 mg CTA; 0.050 M KP, receiving phase: 0.10 M Na_2_S_2_O_3_.

A series of transport measurements using a membrane with a fixed carrier composition, i.e., 0.050 M KP and 25 mg CTA and a plasticizer volume in the range of 0.5–6.0 cm^3^ per 1.0 g CTA were performed for the quantitative determination of the optimum content of *o*-NPPE plasticizer in the membrane (Figure 5).

The introduction of a plasticizer content of 4.0 cm^3^ or greater per 1.0 g of CTA did not affect the rate of Ag(I) transport through PIM. Dissolution of calixpyrroles into the matrix was observed when there was a high content of *o*-NPPE ether in the polymer inclusion membrane, which resulted in effective transport of Ag(I) ions. Confirmation of such composition can be found in a few studies. For unplasticized CTA membrane, no transport of Ag(I) ions was observed. As the content of plasticizer used in the membrane increased to 4.0 cm^3^/1.0 g CTA for the *o*-NPPE and *o*-NPOE plasticizers (76% and 77 wt.%), the permeability of Ag(I) ions through PIM increased significantly and then began to fall. The membranes with the Cyanex 272 carrier plasticized with *o*-NPOE ether described by Rodriguez et al. [34] exhibited maximal values for the permeability coefficients of indium(III) ions, with a plasticizer content of about 78% wt. In turn, Raut et al. [34] obtained the maximal permeability of cesium(I) ions for CTA membranes containing calix[4]bis-2,3-naphtho-crown-6 and 65% wt. *o*-NPOE. On the other hand, in order to achieve maximum transport efficiency of Pb(II), Zn(II), and Cd(II) ions through PIM with crown or lariat ether as a carrier, Ulewicz [35] applied membranes containing 63% wt. and 68% wt. of the *o*-NPOE. A noticeable decrease in the permeability of membranes with a high plasticizer content might be caused by changes in the physico-chemical properties of the membrane due to plasticizer leakage from the membrane phase to the aqueous phase [36].

**Figure 6:**
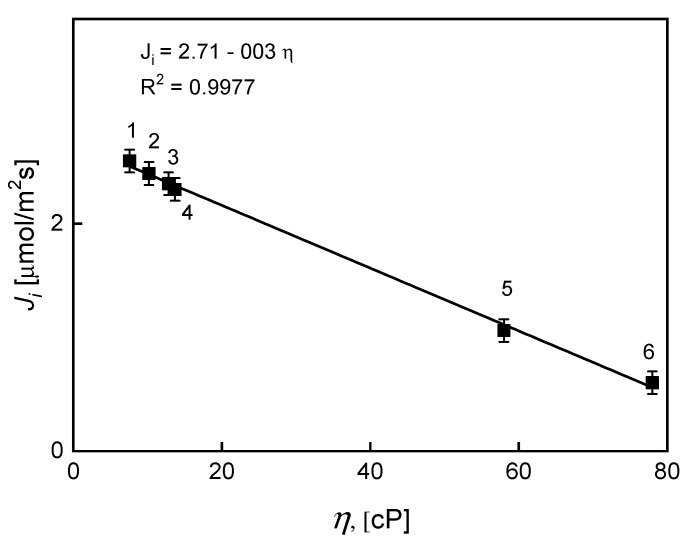
The effect of plasticizer viscosity on the initial fluxes of Ag(I) transport across PIMs. Source phase: 1.0 × 10^−3^ M AgNO_3_ (pH = 4,0); PIMs: 25.0 mg CTA, 4.0 cm^3^ *o*-NPPE (1), TOF (2), *o*-NPOE (3), DOA (4), TCF (5), DOP (6)/1.0 g CTA; 0.050 M KP; receiving phase: 0.10 M Na_2_S_2_O_3_.

Plasticizers such as *o*-nitrophenyl pentyl ether (*o*-NPPE), trioctyl phosphate (TOF), *o*-nitrophenyl-octyl ether (*o*-NPOE), dioctyl adipate (DOA), tricresyl phosphate (TCF), and dioctyl phthalate (DOP) were used at concentrations of 4 cm^3^/1.0 g CTA as the plasticizers of CTA membranes, with the same content of KP (0.10 M). The effect of the plasticizer type on transport through PIM with KP is illustrated in Figure 6.

The linear relationship of the Ag(I) ion transport rate vs. the plasticizers viscosity coefficient was determined using plasticizers with different viscosity coefficients. An increase in viscosity does not favor the diffusion of metal complexes, resulting in a visible decrease in the rate of transport. A decreasing order of the effectiveness of Ag(I) ions permeation was determined using various plasticizers based on the flux value, as follows: *o*-NPPE > TOF > *o*-NPOE > DOA >> TCF >> DOP. *O*-nitrophenyl pentyl ether (*o*-NPPE), and *o*-nitrophenyl-octyl ether (*o*-NPOE). This order showed the relatively best solvents favoring the formation of complexes with Ag(I) as well as the decomplexation reaction, due to the high dielectric constant (ε), which reached a value of 24.

### 2.5. Effect of Temperature and Diffusion

Another factor that might significantly affect the efficiency of metal ion transport through polymer inclusion membranes is the temperature of the process. The activation energy required for the transport process through PIM was determined based on the Arrhenius equation. For this purpose, measurements of Ag(I) ion transport through the PIM containing 0.10 M KP were performed by changing the temperature from 25.0 to 60.0 °C. The flux of metal ion transport in liquid membranes depends on many factors related to the conditions of the experiment, i.e., the temperature, membrane phase viscosity, carrier concentration, pH, and metal concentration in the source phase, and these can be presented by the following equation:

log*J* = A + log*T* − log*η* + log[*H^+^*] + log[KP] + logc*_Ag_*_(*I*)_ (1)

where KP represents calixpyrrole, *η* is the coefficient of the organic phase viscosity in membrane in Cp, *c_Ag(I_*_)_ is the silver concentration in the source phase in M, *T* is the temperature of transport in K, and *A* is an empirical constant.

**Figure 7:**
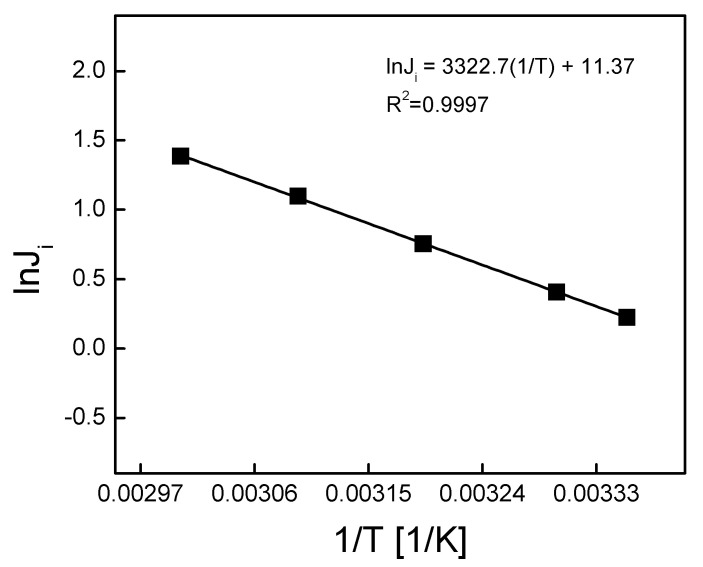
Arrhenius plot of Ag(I) transport across the PIM with calixpyrrole (KP). Source phase: 1.0 × 10^−3^ M AgNO_3_, pH = 4.0; membrane: 4.0 cm^3^ *o*-NPPE/1.0 g CTA; 0.050 M KP, receiving phase: 0.10 M Na_2_S_2_O_3_.

The activation energy of (*E_a_*) was determined based on the relationship of ln(*J*_0_) vs. 1/T and from Equation (2) (Figure 7).

(2) $$J_{i}=\frac{V}{A}\times k\times c_{i}$$

where *J_i_* is the initial flux of metal ions in mol/m^2^s, *V* is the volume of the aqueous source phase in m^3^, and *A* is the area of the membrane in m^2^.

On the basis of the linear regression equation and data presented in Figure 7, the *E_a_* value for the transport process through a membrane immobilized with KP was determined. The value of activation energy determined in the temperature range of 25–60 °C for KP was 27.63 kJ/mol. The value of activation energy was close to the value reported in the literature for alkali metal ion transport through SLM membranes, containing calix[4]arene with four ester groups or calix[4]arene with crown-5 [37]. For the transport of K^+^ and Na^+^ across membranes containing an ester derivative of calix[4]arene, the activation energy values were 60 and 32 kJ/mol, respectively.

The transport process through PIM could be controlled by a silver complex diffusion process in the membrane and did not depend on reactions occurring at the border of the aqueous phase and the membrane. A much smaller activation energy value, i.e., 22.6 kJ/mol, for transport through liquid membranes containing LIX54 as an ion carrier, was obtained by Lazarova [38]. In this case, the transport processes through PIM and SLM were controlled not only by the diffusion process in the membrane but also through chemical reactions occurring in the transport process. For example, transport of tetraphenyl-borate ions through lipid membranes (containing amino acids or their derivatives) resulted in an activation energy (using impedance spectroscopy) corresponding to the stage of diffusion and chemical conversion equal to 38 kJ/mol [39]. It was assumed that values of activation energy lower than 20 kJ/mol indicated diffusive transport, and when (*E_a_*) was higher than 42 kJ/mol, the transport was controlled by a chemical reaction. In cases where the value of activation energy is in the range of 20 to 42 kJ/mol, the transport rate depends on both the diffusion process and chemical reaction [40]. In our case, the flux of Ag(I) transport through polymer inclusion membranes with KP increased as the temperature increased; however, the value of *E_a_* indicated that the process was controlled by the mixed kinetics.

### 2.6. Mechanism of Transport

Diffusion of Ag(I) ions in the membrane could be described using a few transport stages occurring simultaneously, i.e., chemical reactions, metal ion complexation, and decomplexation with the carrier, as well as diffusion of complex or ion carriers in the membrane.

The phenomenon of facilitated transport involves the formation and dissociation of metal complexes with the carrier (KPAg) at the aqueous solution/membrane interface. The carrier (KP) was soluble in the aqueous diffusion layer, and metal ions (Ag) were soluble in the organic phase of the membrane in the form of complexes. The complexation equilibrium was determined using the following equation:

(3) $${\mathrm{KP}}_{\mathrm{org}}+{\mathrm{Ag}}_{\mathrm{aq}}↔{\mathrm{KPAg}}_{\mathrm{org}}$$

Indices _org_ and _aq_ represent the organic phase and the aqueous phase of the membrane, respectively. 

The concentration [KPAg] of complex KPAg at the aqueous solution/membrane interface was expressed by Equation (4):

(4) $${\left[\mathrm{KPAg}\right]}_{i}=K_{ass}\times {\left[\mathrm{KP}\right]}_{i}\times {\left[\mathrm{Ag}\right]}_{i}$$

[KP]*_i_* and [Ag]*_i_* are the concentrations of the carrier and silver ions at the membrane/source phase interface. *K_ass_* is the association (formation) constant of the complex (KPAg); it is also an equilibrium constant of the reaction at the boundary layers of the source phase/membrane. The kinetic stage was determined by the flux (*J*), with Equation (5), derived from Fick’s first law, which meant that the concentration of the complex was negligible at the membrane/receiving phase border.

(5) $$J=(D_{i}/d_{i})\times [\mathrm{KPAg}]$$

where *D_i_* is the diffusion factor in cm^2^·s^−1^, and *d_i_* is the thickness of membrane. The membrane thickness under optimum conditions, i.e., 4.0 cm^3^ *o*-NPPE/1.0 g CTA, was 25 μm.

However, at the interface of the membrane and source phase, [KPAg]*_i_* << [Ag]*_i_*, and, at any moment, the [Ag]*_t_* concentration in the source phase was equal to the [Ag]*_i_* concentration in the diffusion layer of the membrane ([Ag]*_i_* = [Ag]*_t_*). The total concentration of the carrier [KP]_0_ immobilized in the membrane was constant and equal to the sum of the [KP]*_i_* and [KPAg]*_i_* concentrations. [KP]*_i_* and [KPAg]*_i_* were, respectively the concentrations of the carrier and the substrate at the source phase membrane interface.

[KP]_0_ = [KP]*_i_* + [KPAg]*_i_* = [KPAg]*_i_* × [(1 + *K_ass_* × [S]*_i_*/(*K_ass_* × [Ag]*_i_*)] (6)

[KPAg]*_i_* = [KP]*_i_* × *K_ass_* × [Ag]*_i_*/(1 + *K_ass_* × [Ag]*_i_*)] (7)

However, under the initial conditions, the concentration of metal ions was higher than that of the carrier of the source phase/membrane interface; this could be described as [Ag]*_i_* = [Ag]_0_ = *c_i_* and [KPAg]*_i_* ≈ [KP]*_i_*.

Using Equations (5) and (7), the initial value of the stream *J_i_* = (*D_i_*/*d_i_*) × [KPAg]*_i_* was determined through the relationship $J_{i}=(D_{i}/d_{i})\times ({\left[\mathrm{KP}\right]}_{i}\times K_{ass}\times {\left[\mathrm{Ag}\right]}_{i})/(1+K_{ass}\times {\left[\mathrm{Ag}\right]}_{i})$.

Therefore, the relationship of *J_i_* for the initial concentration [KP]*_i_* and *c_i_*, where (*c_i_* >> [KP]*_i_*), was

(8) $$J_{i}=(D_{i}/d_{i})\times ({\left[\mathrm{KP}\right]}_{0}\times K_{ass}\times c_{i})/(1+K_{ass}\times c_{i})$$

In order to determine *D_i_* and *K_ass_*, the relationship shown in Equation (8) was linearized 1/*J_i_* = *f* (1/*c_i_*) by Equation (9):

(9) $$1/J_{i}=(d_{i}/D_{i})\times [(1/{\left[\mathrm{KP}\right]}_{i}\times K_{ass})\times (1/c_{i})+(1/{\left[\mathrm{KP}\right]}_{i})]$$

The *K_ass_* and *D_i_* values were calculated based on the relationship

(10) $$K_{ass}=b/a\;\mathrm{and}\;D_{i}=(d_{i}/b)\times (1/{\left[\mathrm{KP}\right]}_{i})$$

The expression 1/*J_i_* as a function of 1*/c_Ag_*_(*I*)_ was drawn based on the relationship from Equation (9) for the carrier KP, in order to determine *K_ass_* and *D_i_* values.

**Figure 8:**
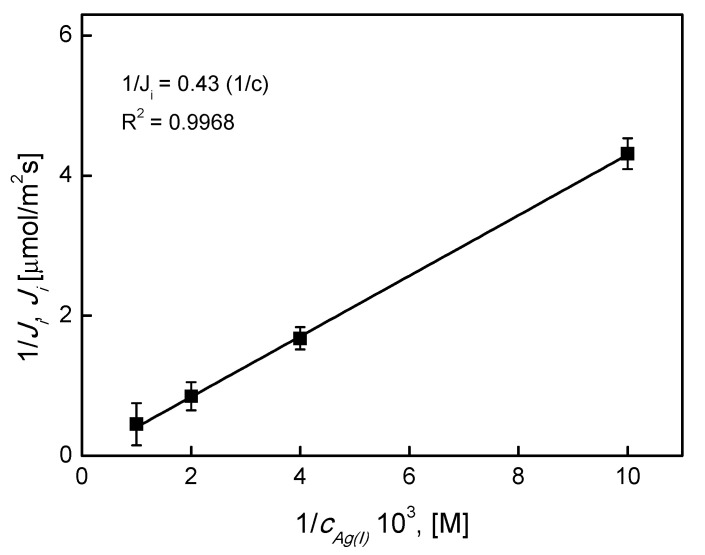
Relationship of 1/*J_i_* vs. 1/ c*_Ag_*_(*I*)_ for Ag(I) transport across a PIM containing KP. Source phase: 10^−4^ M ÷ 10^−3^ M AgNO_3_, pH = 4.0; membrane: 4.0 cm^3^ *o*-NPPE/1.0 g CTA; 0.050 M KP, receiving phase: 0.10 M Na_2_S_2_O_3_.

These results confirmed that the proposed stoichiometry for complex formation with the ion carrier in the organic phase was (M:L = 1:1) The transport rate through the membrane was the stage controlling the rate of metal ion complex transport by diffusion (membrane tortuosity). Values “*a*” and “*b*” determined from the linear regression of metal complexes in the membrane are presented in Figure 8. They were used when applying Equation (10) to determine the apparent diffusion coefficient D and formation constant *K_ass_*.

The *K_ass_* and D values obtained for transport across the PIM with KP were 1.80 and 5.19 × 10^−6^ cm^2^·s^−1^, respectively. It could be concluded that the values of the apparent diffusion coefficient D were about 1 × 10^−6^ for Ag(I) ion transport with different calixpyrrole ligands and were comparable to the literature values [41,42]. The same mechanism was proposed during the transport of sugars [43,44]. The explanation of this phenomenon was that the diffusion process took place by a slight convection movement, which was typical for liquid membranes. Another hypothesis was a transport mechanism with a fixed site carrier system, which was also observed in sugar transport by Smith et al. [45,46]. In our case, the transport mechanism could be described as facilitated transport, with the metal complex diffusion as the controlling stage in the process of Ag(I) ion permeation through the PIM.

The silver-facilitated transport by PIM with the calix[4]pyrrole process occurred according to a co-transport mechanism. The silver ion complex was neutralized by nitrate ions at the interface source phase/membrane. Next, the resulting ion-paired complex diffused across the PIM to the interface membrane/receiving phase, where decomplexation of the metal ion occurred via the formation of a compound with the thiosulfate ion. In the next stage, the ion carrier migrated back to the pores of the plasticizer membrane and the driving process occurred again. The effect of this mechanism was the active transport of silver through the membrane.

### 2.7. PIM Selectivity

PIM selectivity is an effective parameter that allows for the evaluation of the purification degree of a species with respect to others. The selectivity of the membrane system designed for the transport of Ag(I) ions over other transition metals present at equimolar concentrations in three different mixtures in the source phase solution is illustrated in Table 1.

The separation properties of KP were determined in the transport of ions from the receiving phase containing a 5.0 × 10^−4^ M solution of Ag(I) and other metal ion nitrates at a pH of 4.0. In these experiments, PIM containing 0.05 M KP and 4 cm^3^ of *o*-NPPE/1.0 g CTA was used.

The selectivity of these membrane systems was determined with the Ag(I) >> Cu(II) > Pb(II), Ag(I) >> Cd(II)> Zn(II), and Ag(I) >> Zn(II)> Co(II) series. After 4 h, the determined *RF* values for KP were above 92% for Ag(I) and less than 1% for the other metal ions. The values of the selectivity coefficients, due to the low affinity of calixpyrroles to divalent metal ions, were high. These values are provided in Table 1.

The selectivity of silver transport over the other cations was due to the high affinity of the carrier KP for complexation of the Ag(I) ion than the other studied cations [47,48]. Transported Cu(II), Ni(II), Co(II), and Cd(II) ions have smaller ionic radii (0.70–0.95 Å) than silver ions (1.15 Å); therefore, the formation of these complexes are difficult. The silver ion, as a larger cation, was able to coordinate more donor atoms inside the cavity of KP, which led to the formation of more stable complexes. Smaller cations with larger ionic charges tend to undergo a solvation process and their complexes formed with macrocycles are less stable [49]. These high separation factors obtained for the studied metal ions were in contrast with those reported by other authors [50,51,52].

### 2.8. PIM Characterization by SEM

The SEM photomicrographs (Table 2) showed possible degradation of the surface after the process of Ag(I) ion transport.

The comparison of the surface microstructure demonstrated the differentiation in the membrane matrix materials in terms of quantity and sediment distribution, as a result of the phenomena of Ag(I) ion accumulation in the membrane phase. Moreover, the images showed visible roughness of film surfaces. Carriers could crystallize in the membrane.

## 3. Materials and Methods

### 3.1. Reagents

Inorganic chemicals, i.e., AgNO_3_, Cu(NO_3_)_2_, Pb(NO_3_)_2_, Cd(NO_3_)_2_, Ni(NO_3_)_2_, Zn(NO_3_)_2_, Co(NO_3_)_2_, sodium hydroxide, hydrochloric acid, thiourea, sodium thiosulfate, and sodium acetate were of analytical grade and were purchased from POCh (Gliwice, Poland). Organic reagents, i.e., cellulose triacetate (CTA), *o*-nitrophenyl pentyl ether (*o*-NPPE), *o*-nitrophenyl octyl ether (*o*-NPOE), dioctyl adipate (DOA), tricresyl phosphate (TCF), dioctyl (DOP), and dichloromethane were also of analytical grade and were purchased from Fluka (Seelze, Germany) and used without further purification. Aqueous solutions were prepared with double distilled water, with a conductivity of 0.1 µS cm^−1^.

### 3.2. Synthesis

**Figure 9:**
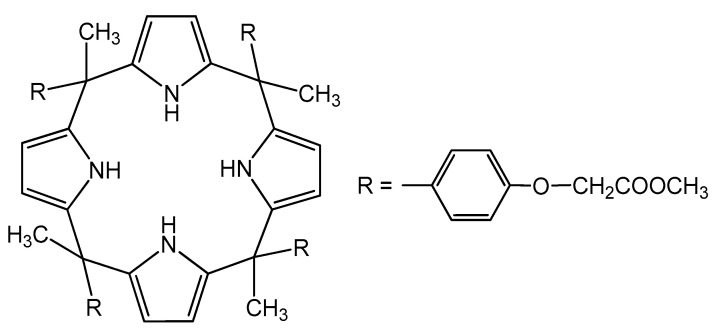
Structures of *meso*-tetra methyl tetrakis-[methyl-2-(4-acetlphenoxy)] calix[4]pyrrole (KP).

The synthesis of *meso*-tetra methyl tetrakis-[methyl-2-(4-acetylphenoxy)] calix[4]pyrrole (KP) (Figure 9) was performed with the reaction of pyrrole with ketones catalysed with acids. The products of such condensation were mainly isomers of four-membered calixpyrrole rings. *Meso*-tetra methyl tetrakis-[methyl-2-(4-acetylphenoxy)] calix[4]pyrrole was obtained from pyrrole and ethyl-2-(4-acetylphenoxy)acetate, following the literature data [53]. To a round-bottomed flask containing pyrrole in dry CH_2_Cl_2_, methyl-2-(4-acetylphenoxy)acetate was added. The mixture was cooled to 0 °C and stirred for 5 min. The reaction mixture was degassed by bubbling with Ar for 10 min. Then, HCl was added dropwise over the mixture for ten minutes, under a nitrogen atmosphere. The resulting solution was stirred at 0 °C for 2 h and then kept at room temperature overnight. The solvent was removed and the crude product was dissolved in ethyl acetate. The solid was washed with water several times and dried with MgSO_4_. Chromatographic purification (silica gel, chloroform/methanol: 1/1) yielded a white solid (80%). The structure of KP was confirmed by ^1^HNMR (Bruker Advance 200, Billerica, MA, USA), (300 MHz, *d*_6_-DMSO, 298 K, ppm): 7.97 (s; 4H, NH); 6.81 (d, 8H, ArH); 6.75 (d, 8H, ArH); 6.01 (d, 8H, PyH); 4.66 (s, 8H, CH_2_); 2.18 (s, 12H, CH_3_); 1.83 (s, 12H, CH_3_).

### 3.3. Preparation of Polymer Inclusion Membranes

A solution of cellulose triacetate (CTA) as the support, *o*-nitrophenyl pentyl ether (*o*-NPPE) as the plasticizer, and calix[4]pyrrole KP (Figure 9) as the ionic carrier was prepared in dichloromethane. The solution was then poured into a 5.0 cm diameter Petri dish, covered with a glass funnel, and then slow evaporation of the solvent occurred overnight. The resulting membrane was separated from the glass plate by immersion in cold water.

### 3.4. Transport Studies

Transport experiments were carried out in a permeation cell in which the membrane film (4.9 cm^2^ effective surface) was tightly clamped between two compartments. In the initial experiments, the aqueous source phase was a 0.0005 mol/dm^3^ AgNO_3_, and the aqueous receiving phase was 0.1 mol/dm^3^ thiourea, sodium thiosulphate, or sodium acetate (50 cm^3^). The Ag(I) transport study was performed in a permeation cell at room temperature (25 °C). The source and receiving aqueous phases were constantly stirred at 600 rpm with motors. Samples (0.5 cm^3^) were collected from an aqueous source and the receiving phases and analyzed to determine the Ag(I) concentration. The acidity of the source and receiving phases was measured by a pH meter (CX-731 Elmetron, pH electrode ERH-136, Hydromet, Poland). The parameters describing transport such as the rate constant *k*, permeability *P*, and initial flux *J_i_*, were calculated from the following equations [54]:

(11) $$\ln\left(\frac{c}{c_{i}}\right)=-kt$$

(12) $$P=-(\frac{V}{A})k$$

(13) $$J_{i}=Pc_{i}$$

where *c* is the metal ion concentration (mol/dm^3^) in the source phase at a given time, *c_i_* is the initial Ag(I) concentration in the source phase, *k* is the rate constant (s^−1^), *t* is the transport time (s), *V* is the volume of the aqueous source phase, and *A* is the area of the membrane.

To describe the efficiency of metal ion removal from the source phase, the removal factor (*RF*) was calculated as

(14) $$RF=\left(\frac{c_{i}-c}{c_{i}}\right)100\%$$

The selectivity coefficient (*S*) was defined as the ratio of initial fluxes for M1 and M2 metal ions, respectively:

(15) $$S=\frac{J_{i},M1}{J_{i},M2}$$

The metal ion concentrations were measured by flame atomic absorption spectrometry (Solar 939, Unicam, Thermo Fisher Scientific, Waltham, MA, USA). The reported values corresponded to the average values of three replicates; the standard deviation observed was within 2%.

### 3.5. Characteristic of PIM

A 5 KV scanning electron microscope (SEM) (Quanta 3D FEG, FEI Company, Hillsboro, OR, USA) (Hitachi S4500) was used to elucidate the membrane morphology. Membrane samples were prepared by freezing under liquid nitrogen (70 K) and rapid fracturing, resulting in the formation of a clean break fracture image, which was used to view the cross-section. The samples were mounted with conductive glue to metal stubs with the fractured edge up, and then they were coated with gold by sputtering. These samples were then viewed in the SEM at a magnification of around 50 µm.

## 4. Conclusions

Successful transport of Ag(I) through a PIM containing KP was controlled by the modification of process parameters such as the pH of the source solution, the membrane, the composition of the source and the receiving phases, as well as the metal ion concentration in the source phase. We showed that the polymer inclusion membrane (CTA/*o*-NPPE) containing KP effectively transported Ag(I) from aqueous solutions at a pH of 4.0 into an 0.1 M Na_2_S_2_O_3_ aqueous receiving solution. This process was very fast and efficient when the metal concentration in the source aqueous phase was below 10^−3^ M. Optimized transport conditions showed that the flux of Ag(I) ions through the PIM (J = 2.45 µmol/m^2^·s) was higher than the values reported in other studies, regarding silver transport by liquid membranes containing macrocyclic carriers. The highest rate of Ag(I) transport across the plasticizer membrane was observed with 0.05 M of the carrier. Increasing the amount of *o*-NPPE in the membrane caused an increase in membrane viscosity. Increasing the amount of plasticizer above 4.0 cm^3^/1.0 g CTA, resulted in a reduction in the rate of transport across the membrane.

The description of Ag(I) transport kinetics across the PIM was based on the mechanism involving the formation of a complex carrier–metal ion boundary layer at the membrane interface, and the diffusion of this complex via the liquid pores of the plasticizer membranes. The ion transport mechanism was confirmed by the *K_ass_* and D values to influence the reaction of complex formation, and its diffusion through the organic phase of the PIM. The low association constant values (*K_ass_* = 1.80) determined in the transport process, as well as the relatively high diffusion coefficient value (D = 5.19 × 10^−6^ cm^2^·s^−1^) might be the reason for the fast transport rate observed for carrier-mediated transport of Ag(I) ions through KP immobilized into the CTA membrane.

Due to their excellent properties, PIMs with immobilized KP as the carrier might be a promising approach for selective extraction of Ag(I) from a mixed solution of Cu(II), Pb(II), Cd(II), Ni(II), Zn(II), and Co(II), e.g., copper smelting wastewater.

Using the microscopic method, it was shown that the membrane containing the ester derivative calix[4]pyrrole was rough.

## Figures and Tables

**Table 1:** The initial fluxes (*J_i_*) and selectivity orders and coefficients (S_M1/M2_) obtained in the transport of Ag(I), Cu(II), Pb(II), Cd(II), Ni(II), Zn(II), and Co(II) across PIMs. Source phase: 5.0 × 10^−4^ M of each metal ion; membrane: 4.0 cm^3^ *o*-NPPE/1.0 g CTA; 0.050 M KP; receiving phase: 0.10 M Na_2_S_2_O_3_ aqueous solution.

Metal Ions	*RF*, %	*J_i_*, µmol/m^2^s	Selectivity Orders Selectivity Coefficients, *S*
Ag(I)	92	2.10	Ag(I) >> Cu(II) > Pb(II) 30 210
Cu(II)	0.5	0.07	
Pb(II)	0.4	0.01	
Ag(I)	95	2.14	Ag(I) >> Cd(II) > Ni(II) 71 214
Cd(II)	0.15	0.03	
Ni(II)	0.05	0.01	
Ag(I)	94	1.99	Ag(I) >> Zn(II) > Co(II) 132 199
Zn(II)	0.03	0.015	
Co(II)	0.02	0.01	

**Table 2:** SEM images before and after the transport of Ag(I) ions across a PIM containing KP (magnification 50 µm).

Before Transport Ag(I)	After Transport Ag(I)